# The complete swine olfactory subgenome: expansion of the olfactory gene repertoire in the pig genome

**DOI:** 10.1186/1471-2164-13-584

**Published:** 2012-11-15

**Authors:** Dinh Truong Nguyen, Kyooyeol Lee, Hojun Choi, Min-kyeung Choi, Minh Thong Le, Ning Song, Jin-Hoi Kim, Han Geuk Seo, Jae-Wook Oh, Kyungtae Lee, Tae-Hun Kim, Chankyu Park

**Affiliations:** 1 Department of Animal Biotechnology, Konkuk University, 263 Achasan-ro, Gwangjin-gu, Seoul, 143-701, South Korea; 2 Division of Animal Life Science, Konkuk University, Seoul, South Korea; 3 Animal Genomics and Bioinformatics Division, National Institute of Rural Development Administration, Suwon, South Korea

**Keywords:** Olfactory receptor, Pigs, Olfaction, OR genes

## Abstract

**Background:**

Insects and animals can recognize surrounding environments by detecting thousands of chemical odorants. Olfaction is a complicated process that begins in the olfactory epithelium with the specific binding of volatile odorant molecules to dedicated olfactory receptors (ORs). OR proteins are encoded by the largest gene superfamily in the mammalian genome.

**Results:**

We report here the whole genome analysis of the olfactory receptor genes of *S. scrofa* using conserved OR gene specific motifs and known OR protein sequences from diverse species. We identified 1,301 OR related sequences from the *S. scrofa* genome assembly, Sscrofa10.2, including 1,113 functional OR genes and 188 pseudogenes. OR genes were located in 46 different regions on 16 pig chromosomes. We classified the ORs into 17 families, three Class I and 14 Class II families, and further grouped them into 349 subfamilies. We also identified inter- and intra-chromosomal duplications of OR genes residing on 11 chromosomes. A significant number of pig OR genes (n = 212) showed less than 60% amino acid sequence similarity to known OR genes of other species.

**Conclusion:**

As the genome assembly Sscrofa10.2 covers 99.9% of the pig genome, our analysis represents an almost complete OR gene repertoire from an individual pig genome. We show that *S. scrofa* has one of the largest OR repertoires, suggesting an expansion of OR genes in the swine genome. A significant number of unique OR genes in the pig genome may suggest the presence of swine specific olfactory stimulation.

## Background

Insects and animals can recognize the world around them by detecting thousands of chemical odorants. In mammals, odorant molecules are detected by olfactory receptors (ORs), which are part of the G-protein-coupled receptor superfamily of proteins having seven transmembrane domains. This superfamily was first discovered in rodents about two decades ago [1]. Olfaction is a complicated process; it begins in the olfactory epithelium with the specific binding of volatile odorant molecules to dedicated ORs expressed by olfactory sensory neurons (OSNs) [2-5].

OR proteins are encoded by the largest gene superfamily in the mammalian genome. Using the available genome sequences, several studies have been conducted to elucidate OR subgenomes in species such as mice [6-9], humans [10-13], dogs and rats [14-16], and other vertebrates [14,17-19]. OR gene families can be grouped into the following two classes: the fish-like Class I ORs consisting of 17 families and the tetrapod-specific Class II ORs consisting of 14 families [18]. The number of functional OR genes ranges from less than 100 in some fishes including fugu (n = 44) and tetraodon (n = 42) [20] to ~1,200 in rats. A significant number of OR genes have pseudogenes, and the fraction of OR pseudogenes ranges from less than 20% in the opossum to more than 50% in humans or platypus [14,17]. Interestingly, in spite of the large number of genes that make up the OR subgenome, most OR neurons express a single gene and in fact, even just a single allele [1,21].

Pigs are an attractive animal model to study olfaction and its influence on animal behavior because of their agricultural importance and their strong reliance on their sense of smell in various behavioral contexts. The characterization of the swine OR gene repertoire is necessary to better understand the underlying biology of olfaction in pigs. In addition, the comparison of OR gene repertoires and the abilities to smell among evolutionarily important animals is an interesting subject. In this study, we analyzed the pig genome assembly Sscrofa10.2, constructed by the Swine Genome Sequencing Consortium (SGSC), to characterize OR genes in pigs. We report here the nearly complete porcine olfactory subgenome. In addition, we classified the pig OR genes into families and compared OR gene repertoires of humans, dogs, mice, and pigs.

## Methods

### Detection of OR genes from the pig genome

The swine draft genome sequences (Sscrofa10.2) were retrieved from the National Center for Biotechnology Information (NCBI). A translated basic local alignment search tool (TBLASTN) search was performed to identify regions containing OR related sequences that had at least two of the following conserved motifs: MAYDRYVAIC (TMIII), KAFSTCASH (TMVI), and PMLNPFIY (TMVII), or their variants with less than 50% of sequence difference from the conserved motifs. From the identified regions, we selected the sequences in the region one kilobase (kb) upstream and downstream of the BLAST matches. From the analysis, we identified 1,644 OR candidate sequences that were 2 kb in length and translated to amino acid sequences in all six frames. Then, we retrieved 24,809 OR protein sequences from 222 species from NCBI and performed a protein BLAST (BLASTP) analysis against the translated OR candidate sequences to determine the positions of the start and stop codons of the open reading frames (ORFs) on the basis of structural similarity to known OR proteins. For sequences that deviated from the sequences of reported OR proteins, the methionine and stop codon most similar in sequence context to those of the coding sequences of known OR proteins were selected as the start and end of the coding regions. We again performed TBLASTN analysis against the 1,644 sequences to evaluate the presence of all four conserved motifs [GN, MAYDRYVAIC (TMIII), KAFSTCASH (TMVI), and PMLNPFIY (TMVII)]. The candidate sequences were considered “functional ORs” if they were at least 300-amino acid long without any interrupting stop codons and/or frameshifts within the ORFs, “OR pseudogenes” if they were at least 300-amino acid long but contained stop codons or frameshifts within the ORFs, and “partial ORs” if they were shorter than 300 amino acids in length but matched the sequences of the known OR genes. Sequences similar to non-OR G-protein-coupled receptors or partial sequences were removed from our analyses, leaving 1,301 OR genes (including pseudogenes).

### Phylogenetic analysis and classification

The nucleotide sequences of 3,511 OR genes from human (457), mouse (908), dog (845), and pig (1,301, 1644 putative ORs minus 343 partial ORs) were combined and aligned together using CLUSTALW [22]. An unrooted phylogenetic tree was constructed after 1,000 rounds of bootstrapping. The tree was used for classifying OR gene families and subfamilies. Pig OR sequences that did not form a cluster with any reference ORs from the other three species were additionally classified using a sequence similarity matrix (data not shown) in which 40% and 60% amino acid similarity were used as the thresholds to distinguish between families and subfamilies, respectively, as previously described [23].

### OR gene nomenclatures

For naming pig OR genes, we followed the OR gene classification system described by Glusman *et al.*[23]. Functional pig OR genes were named “sORmXn” whereas pseudogenes were named “sORmXnP”, where “s” stands for *S. scrofa*, “OR” is the root name indicating an olfactory receptor, “m” is an integer representing the family that the gene belongs to, “X” is a single letter denoting the subfamily of the gene, and “n” is an integer representing an individual family member. The names of the pig OR sequences were devised on the basis of on their phylogenetic relationships. For example, sOR1A1 is an OR gene of family 1, subfamily A, and is the first member of this subfamily. In the case of pseudogenes, a name such as sOR7E12P indicates an OR pseudogene of family 7, subfamily E, that is the twelfth member of this subfamily. Duplicated genes with the exact same coding sequences were indicated by adding the suffix A, B, or C at the end of their names, i.e., sOR51N3A and sOR51N3B.

### Identification of pig specific OR genes

Multispecies OR gene clustering analysis was performed with OR protein sequences from humans, dogs, mice, and pigs using the OrthoMCL 3 software [24], in order to group them on the basis of their sequence similarity and divergence. In total, 706 clusters were formed from 3,511 sequences. The cutoff value for a cluster was 60% similarity at the level of the protein sequence, resulting in sequences with greater than 60% similarity being clustered together regardless of the species of origin.

### Detection of conserved motifs and patterns

To detect conserved motifs in predicted OR protein sequences, sequence logos were generated from an alignment of functional OR gene sequences using the WebLogo program [25]. The PRATT [26] program from the Pattern Discovery Platform [27] was used to define pig OR-specific patterns with the criteria listed in Additional file 1.

## Results

### Composition of the pig OR gene repertoire

**Figure 1:**
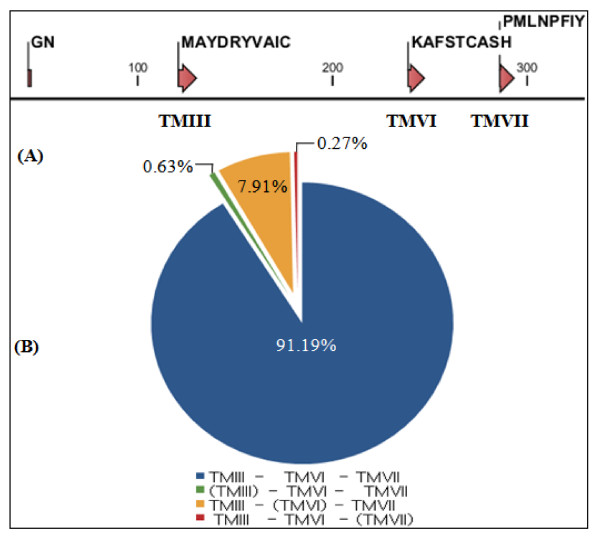
**Conserved olfactory receptor (OR) specific motifs used to identify OR genes in the pig genome, and the frequency of sequences with or without these motifs. (A)** The amino acid sequences of the OR specific motifs are shown. The numbers indicate the positions of amino acids. TM, transmembrane domain. **(B)** Proportional distribution of the 1,301 functional and pseudo OR amino acid sequences identified by their OR motif containing patterns. The motifs within parentheses were absent. The absence or presence of the GN motif was not indicated.

The four motif sequences, GN, MAYDRYVAIC, KAFSTCASH and PMLNPFIY, which are common to mammalian OR genes were used to search the full repertoire of ORs in the pig genome (Figure 1A). We identified 1,301 OR gene-related sequences with lengths of 900–1,000 base pairs (bp). We also analyzed their ORFs and grouped them into the following two categories: functional and pseudo genes. In total, 1,113 OR sequences were identified as functional and 188 were identified as pseudogenes. Among the identified functional genes and pseudogenes, 91.19% of the sequences contained all three OR domains and the rest were missing one of the conserved motifs (Figure 1B). For the GN motif, the presence of the motif was difficult to evaluate because the motif was defined by only two amino acids and may also have sequence variations. Therefore we did not include the result.

### Chromosomal distribution of OR genes in the pig genome

**Figure 2:**
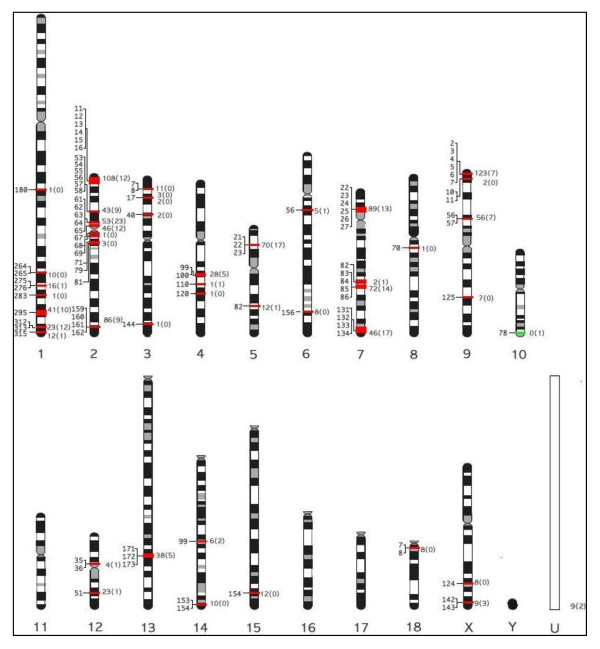
**Chromosomal distribution of pig OR genes.** Pig OR genes were mapped to 46 regions across 16 chromosomes. The number of functional and pseudo OR genes at each cluster is indicated to the right of the chromosomes without and with parentheses, respectively. Clusters with and without functional OR genes are indicated by red and green lines, respectively. The position of each cluster is shown to the left of the chromosomes in megabases (Mb). The names of clusters indicate the chromosome number and distance (Mb) from the top of the chromosome, i.e., the cluster 10–28, positioned at 28 Mb region of chromosome 10. “U” indicates a group of sequences with no chromosome assignment in the pig genome assembly Sscrofa10.2. Chromosome figures were modified from Rothschild *et al.*[28].

The locations of the OR genes were analyzed on the basis of their relative positions in the pig genome by grouping them into positional regions according to their positional proximity. If the coding sequences of the OR genes were more than one megabase (Mb) apart, they were considered to be present on different regions. Of the 1,301 functional genes and pseudogenes, 1,290 were mapped to 46 different chromosomal regions across 16 pig chromosomes and the remaining 11 were located on chromosome U, which contains unmapped sequences (Figure 2). Except for chromosomes 11, 16, 17, and Y, which were devoid of OR genes, all the other chromosomes contained one to 406 OR genes (Table 1). Chromosome 2 had the largest number of OR genes (341), followed by chromosomes 7, 9, and 1. Accordingly, chromosome 2 contained the largest number of OR subfamilies with 121 subfamilies, while only a single subfamily was present on both chromosomes 8 and 10 (Table 1).

**Table 1:** Composition of olfactory receptor genes for each pig chromosome

**Chromosome number**	**No. of functional genes**	**No. of pseudogenes (%)**	**Total no. of OR genes**	**No. of subfamilies**
1	104	24 (19)	128	47
2	341	65 (16)	406	121
3	19	0 (0)	19	8
4	30	6 (17)	36	21
5	82	18 (18)	100	22
6	13	1 (7)	14	4
7	208	45 (18)	253	61
8	1	0 (0)	1	1
9	188	14 (7)	202	72
10	0	1 (100)	1	1
11	0	0	0	0
12	27	2 (7)	29	8
13	38	5 (12)	43	16
14	16	2 (11)	18	8
15	12	0 (0)	12	2
16	0	0	0	0
17	0	0	0	0
18	8	0 (0)	8	3
X	17	3 (15)	20	11
Y	0	0	0	0
U	9	2 (18)	11	10
Total	1,113	188(14)	1,301	

Note: In the case of the absence of both OR functional genes and pseudogenes, the pseudogene % was not indicated.

We observed extensive variations in the number of OR genes at individual OR gene clusters from one to 123 OR genes per locus/cluster (Table 2). Due to the presence of a large number of OR genes in the genome, the number of pseudogenes was also high (n = 188). The percentage of pseudogenes varied among clusters and ranged from 0 to 100% (Table 1). Of the 46 OR gene clusters, the locus “10-78” was the only OR gene locus that had only one pseudogene, while the other 45 clusters had at least one functional gene (Table 2). In the current swine genome assembly Sscrofa10.2, 11 OR genes (nine functional genes and two pseudogenes) were located on unmapped contigs without any chromosome information. Complete information on the distribution of all OR functional genes and pseudogenes in the pig genome is detailed in Additional file 2.

**Table 2:** Analysis of the number of functional olfactory receptor genes and subfamily distribution per cluster

**Functional OR genes per cluster**^**1**^		**Clusters per subfamily**^**2**^		**Subfamilies per cluster**^**3**^	
**No. of OR genes**	**No. of clusters**	**No. of clusters**	**No. of subfamilies**	**No. of subfamilies**	**No. of clusters**
0	1	1	275	1	12
1	7	2	54	2	7
2	4	3	15	3	3
3	2	4	2	4	2
4	1	5	3	5	2
5	1			6	2
6	1			7	1
7	1			8	1
8	3			10	2
9	2			11	1
10	2			16	1
11	1			18	3
12	3			20	1
16	1			21	2
23	2			23	1
28	1			25	1
38	1			31	1
41	1			38	1
43	1			39	1
46	2			52	1
53	1				
56	1				
70	1				
72	1				
86	1				
89	1				
108	1				
123	1				

^1^Number of OR gene clusters with 0 to 123 functional OR genes.

^2^Number of subfamilies whose members are encoded at one to five clusters.

^3^Number of clusters that encode members of one to 52 subfamilies.

### Classification of OR gene repertoires

Understanding the diversity of OR genes is important for elucidating the differences in their functional responses to various odorants. ORs with more than 60% identity in protein sequence are suggested to recognize odorants with related structures [29,30]. To evaluate the diversity in the OR gene repertoire of pigs, the identified pig OR genes were classified into families and subfamilies according to the results of phylogenetic analyses (data available upon requested) and their sequence similarity. Then, the results obtained after the classification were compared with those previously obtained for from humans, dogs, mice, and rats [9,13,16]. Our analysis showed that the pig OR repertoire comprises 17 families and 349 subfamilies; this repertoire is largest among the known repertoires of mammals (Additional file 3). This suggests that compared to other species, pigs may have a more sophisticated system to sense smell and may be able to distinguish more diverse odorants. Although humans and dogs have relatively large number of OR subfamilies (300 each), humans have a higher pseudogene frequency (52%) than pigs, and dogs have a lower number of functional genes (n = 872) than pigs (n = 1,113). This supports the idea that the functional complexity of the pig olfactory system could be attributed, in part, to genetic complexity. Similar to the OR genes of other mammals, pig OR genes could also be classified into two classes, with three Class I families and 14 Class II families (Additional file 3).

The number of OR genes belonging to each subfamily may represent the importance of the specific subfamilies for the species, as the OR gene subfamilies that are important for the survival of the species are likely to expand in the genome through evolution. Therefore, we counted the number of ORs in each subfamily (Additional file 4). The size of pig OR subfamilies was extremely variable with one to 52 OR genes per subfamily. While most subfamilies had one to six members, six subfamilies had more than 20 genes each. The most common type of subfamily comprised only a single OR gene, accounting for 146 subfamilies. In contrast, subfamily sOR6A consisted of 52 genes (data not shown).

### Distribution of OR subfamilies within the OR gene clusters

To study the possible associations between the subfamily structure and the chromosomal organization of OR genes in pigs, the chromosomal locations of all OR gene members of the 349 pig OR subfamilies were analyzed (Table 1). The largest OR cluster in the pig genome was the cluster “9-2” on chromosome 9, which contained 123 OR genes making up 52 subfamilies. We observed that 275 (78.8%) subfamilies were encoded by genes at a single chromosomal cluster, suggesting possible functional similarities among OR genes within a cluster. When we determined the subfamily composition of individual OR gene clusters, the number of subfamilies within a cluster ranged from one to 52 (Table 2). About 26% (12/46) of the OR clusters encoded only one OR subfamily, while 74% of clusters (34/46) encoded OR genes of more than two subfamilies. The general characteristics of the OR subgenome including the number of functional OR genes within a cluster, the number of clusters within a subfamily, and the number of subfamilies within a cluster in the pig (Table 2) were consistent with those reported for other species such as mouse and human [9,13].

### Analysis of OR gene duplication

Gene duplication plays an important role in establishing the biological characteristics or diversity of organisms during evolution [31]. From our analysis to identify OR genes in the pig genome, we found 100% identical coding sequences of OR genes that mapped to different regions in the pig genome. Further analysis showed that the sizes of duplications ranged from 1.1 to 120 kb (data not shown). Duplicated OR genes were found for both functional genes (n = 166) and pseudogenes (n = 22) (Additional file 5), although most of the duplications were of functional genes. There are 80 functional and 11 pseudo genes that have one identical copy each, making 160 and 22 OR genes in total, and two OR genes sOR7A6[ABC] and sOR5AT1[ABC] were found three times each in the pig genome assembly Sscrofa10.2 (Additional file 6). In total, 93 duplication events consisting of 87 intra- and six inter-chromosomal duplications (data not shown) were observed at 11 chromosomes with duplication of two to 41 genes depending on the chromosome (Additional file 5). The most frequent duplication pattern was the presence of two identical OR coding sequences in the genome (Additional file 6). However, we also were not able to entirely exclude the possibility that some of these duplications might result from the errors in the genome assembly. Although we reexamined the partial or duplicated OR genes with respect to assembly issues such as locations in the contigs and relationship between individual members of identical duplicates, we did not find any logical evidences to support that a part of partial or duplicated OR genes were caused by assembly errors.

### Patterns of characteristic amino acid motifs in pig OR proteins

Using the criteria in Additional file 1, we performed a pattern discovery analysis for pig OR genes. Table 3 shows five motif patterns identified from four conserved transmembrane domains of pig OR genes, TMII, TMIII, TMVI and TMVII, which are similar to those reported from other species including dogs [16], rats [16], and humans [13] except for minor differences at variable amino acid sites. Analysis of the similarities and differences in conserved OR transmembrane motifs among different species could elucidate the functional importance of each site within the motifs.

**Table 3:** The representative amino acid patterns of the conserved transmembrane motifs of pig, dog and rat OR genes

**Pattern no.**	**Transmembrane domain**	**Pattern**
Pig	TMII	H-X-P-M-Y-F-F-L-X-[NS]-L-S-[FL]-[AV]-D
1		
2	TMIII	L-X(2,3)-M-[AV]-Y-D-[RS]-F-[LV]-A-I_C-H-P-L-H-Y
3	TMIII	L-X(2,4)-M-[AGS]-X-D-X(2,3)-A-[IV]-X(2)-[LP]-[FIL]
4	TMVI	K-A-[FL]-S-T-C-X-S-H-L-X-V
5	TMVII	P-M-[LM]-N-P-F-[IV]-Y-[NS]-L-X-N-[KR]-[DN]
Dog		
1	TMII	P-M-Y-X-[FL]-L-X(2)-[FL]-[AMS]-X(2)-[DE]
2	TMIII	L-X(3)-M-X(0,1)-Y-X-[FLR]-[LY]-X(2)-[FILV]-[ACS]
3	TMIII	L-X(1,3)-M-X-[FILY]-D-R-X(2)-A-[IV]-[CS]-X-P-L-X-[HY]-X(3)-[ILM]
4	TMVI	K-X-[FL]-[AGHNST]-T-C-X-[AS]-H-X(3)-[AIV]
5	TMVII	N-P-[FILMV]-[IV]-Y-[AGST]-[AILMV]-[KR]-X(2)-[DEKQ]
Rat		
1	TMII	L-[HKNQR]-X-P-M-[FY]-X-[FIL]-L-X(2)-L-X(3)-[DEY]
2	TMIII	M-[AS]-[FLY]-D-R-[FHY]-[AILMV]-A-[IV]-X(2)-P-L-X-[HY]-X(3)-[FILMV]-[DGHKNPRST]
3	TMV	S-Y-X(2)-I-[FILV]-X-[AST]-[FIV]
4	TMVI	K-X-[FILMV]-X-T-C-X-[ACPST]-H-[FILMV]-X(2)-[FILMV]
5	TMVII	P-X-[LMV]-N-P-[FILMV]-X-Y-[ACGST]-X-[KNR]-X-[KNQRT]-[DEKPQ]-[FILMV]

Note: The pattern for dogs and rats was taken from Quignon *et al.*[16]. [XYZ] means X or Y or Z. The lower case letter “X” can be used as a pattern element to denote any amino acid. X(m) is equivalent to the repetition of X exactly m times. X(m,n) is equivalent to the repetition of X exactly k times for any integer k satisfying: m ≤ k ≤ n.

### Potential odorant specificity of OR subfamilies

To identify potential target specificity of pig OR subfamilies in odor perception, we compared the amino acid sequences of the 1,113 translated pig OR genes to those of other species with previously described information on odorant specificity, including two human ORs [32,33] and 20 mouse ORs [29,30,34-38]. From the analysis, we found that 18 pig ORs matched ORs from other species with known specificity with at least 60% sequence identity, suggesting that these ORs may share similar olfactory specificities (Table 4). There were three mouse ORs, Olfr672, Olfr586 and Olfr545, showing less than 60% sequence similarity to pig ORs and they are known to sense *n*-aliphatic acids, *n*-aliphatic alcohols, *n*-aliphatic dicarboxylic acids, and (−) citronellal. In addition, our analysis also showed that no pig OR has sequence similarity to OR3A1; this human OR is known to perceive helional, which has sweet and hay-like smell.

**Table 4:** Potential associations between pig olfactory receptor gene clusters and odorant recognition

**Pig OR cluster**	**Mouse and human ORs with known odorant recognition**^**1**^	**Pig ORs with sequence similarity**	**Amino acid sequence identity( %)**	**Recognized odorant(s)**	**Perceived odor**
9-4	Olfr2	sOR6T9	90	*n*-aliphatic aldehydes	Fatty
9-4	Olfr690	sOR52I8	89	*n*-aliphatic acids/alcohols	As above
12-51	OR1D2	sOR1N1	87	Bourgeonal	Lily of the valley
4-99	Olfr16	sOR10D1	85	Lyral	Lemony, green
7-82	Olfr49	sOR6I1	85	(−) citronellal	Lemon
7-84	Olfr749	sOR11A6	85	*n*-aliphatic acids	Rancid, sour, sweaty, fatty
9-5	Olfr653	sOR52J1	85	*n*-aliphatic acids/alcohols	As above
9-5	Olfr642	sOR51C2	83	*n*-aliphatic acids	As above
9-57	Olfr151	sOR8O1	81	Acetophenone	Floral/woody
2-159	Olfr73	sOR5I2	80	Eugenol	Spicy
9-2	Olfr480	sOR5AA3	79	*n*-aliphatic alcohols	Herbal, woody, orange, rose
9-4	Olfr661	sOR53B4	79	*n*-aliphatic acids/alcohols	As above
9-6	Olfr69	sOR52D3	79	*n*-aliphatic acids/alcohols	As above
1-295	Olfr74	sOR1L2	74	Ethyl vanillin	Vanilla
2-14	Olfr154	sOR5T3	74	2-Heptanone	Fruity
1-295	Olfr50	sOR1L7	73	I-carvone	Spearmint, caraway
9-4	Olfr683	sOR53A1	73	*n*-aliphatic acids/alcohols	As above
3-8	Olfr56	sOR2N1	60	Limonene	Lemon
9-4	Olfr672	sOR52I10	57	*n*-aliphatic acids	Rancid, sour, sweaty, fatty
9-5	Olfr586	sOR51V2	52	*n*-aliphatic acids	As above
9-6	Olfr545	sOR52H3	42	*n*-aliphatic dicarboxylic acids	
-	OR3A1	-	-	Helional	Sweet, hay-like

Note: A dash (−) indicates the absence of corresponding pig ORs. The order of pig OR clusters was based on the amino acid sequence identity (4th column).

## Discussion

ORs in mammals are encoded by several hundreds to many thousands of genes in the genome, which together form the OR subgenome [7,9,10,13,15,16,18,19]. With the availability of whole genome sequence information, several studies have been carried out to characterize the OR subgenomes of vertebrates [9,13,15,16,18,19,39] in an attempt to better understand the underlying biology of olfaction. In this study, we analyzed the current genome assembly of *S. scrofa* using conserved OR motifs and 24,809 OR protein sequences available from NCBI. We also identified and characterized 1,301 OR related sequences and their genomic distributions. Our study, as the first analysis of the OR gene repertoire in artiodactyla, shows the presence of similarities and differences in the genetic make-up between the pig OR system and that of other animals.

The percentage of OR pseudogenes in the OR subgenome could be an important factor in determining the actual size of the OR repertoire and the number of OR genes present in the genome. Our analysis shows that the percentage of OR pseudogenes in the pig genome is 14%, which is the lowest reported fraction of pseudogenes in any species followed by dogs and rats (Table 5). Pigs and rats have the largest functional OR repertoire with 1,113 and 1,201 genes, respectively. It is interesting to speculate that the olfactory capacity of pigs and rats could be superior to that of dogs, which have 872 functional OR genes, when only gene numbers but not the anatomical difference of olfactory system are considered.

**Table 5:** Differences in the frequencies of functional olfactory receptor genes among different species

**Species**	**Number of functional genes**	**Number of pseudogenes**	**Percentage of functional gene**
Pig	1,113	188	86
Rat	1,201	292	80
Dog	872	222	80
Mouse	1,037	354	75
Zebrafish	102	35	74
Human	388	414	48
Frog	410	478	46
Pufferfish	44	54	45
Chicken	82	476	15

Note: Except for pig, data were from Niimura and Nei [40].

The prevalence of pseudogenes in humans and nonhuman primates has been described in several studies as characteristic of these lineages [4,41-44]. Because of the anatomical and physiological similarity between pigs and humans, the importance of pigs as biomedical models or donors for human xenotransplantation has recently been suggested [45]. On the other hand, the genetic system of olfaction could be the one of the major differences between humans and pigs; this is consistent with the concept of primates as visual mammals with reduced olfaction [46]. Although detailed anatomical and functional studies on the olfactory system of pigs are not available, the general behavior of pigs and the size of the genetic content responsible for olfaction in pigs support the hypothesis of olfactory expansion in the pig.

When we compared the structural characteristics of OR gene clusters between pigs, humans, mice, rats, and dogs, we did not observe any distinctive trends or patterns that reflected the size of the OR gene repertoire (Additional file 7). However, the number of OR genes per cluster was related to the size of the OR gene repertoire, indicating that an increase in OR gene numbers in pigs during evolution was not due to an increase in the number of OR clusters, but more likely due to an increase in gene numbers within clusters. The number of nonfunctional OR clusters consisting of only OR pseudogenes without functional genes was limited to only one locus in the pig genome, while 13 such clusters were identified in humans [13].

MHC haplotypes and olfaction have been suspected to be related [47]. Therefore, we determined the number of OR genes that were located on the same chromosome as the MHC region in humans, dogs, mice, rats, and pigs. While the number of OR genes on chromosome 7, which contains the MHC region in pigs, was very high (n = 253), the distribution of OR genes on the MHC containing chromosomes in other species was much lower than that of the pig (data not shown). Further evaluation of the physical distance between OR genes and the MHC region among five species showed that these clusters were not always physically proximal to each other. Especially in dogs, no ORs were found near the MHC region. Although functional relationships may present between OR and MHC molecules, our analysis suggest that the physical linkage between OR clusters and MHC regions may not be strong to all species.

**Figure 3:**
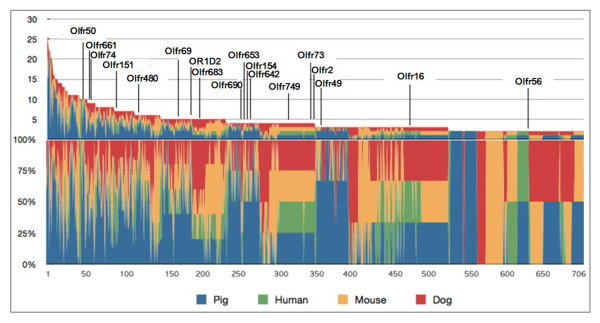
**Comparison of OR gene similarity among humans, dogs, mice, and pigs by clustering analysis of OR genes on the basis of their amino acid sequence similarity.** The names of ORs with known chemical specificity from humans and mice were indicated above the graph. The Y-axis of the upper graph shows the number of OR genes in each cluster ranging from two to 26 genes. The X-axis of both the upper and lower graphs indicates the cluster number, with 706 clusters. The Y-axis of the lower graph indicates the percentage of OR genes of each species within the cluster. The OR genes of different species are indicated by different colors. See the additional file 7 for the uncompressed original image of Figure 3.

To understand the evolutionary relationships between OR genes from pigs, humans, mice, and dogs, we combined 3,511 OR gene sequences from these four species and performed clustering according to their protein sequence similarity (Figure 3). Using a cutoff of more than 60% sequence identity to group sequences together into a single cluster, 706 clusters were generated according to sequence similarity between pigs, humans, mice, and dogs. Intra-species OR subfamily genes that have more than 60% sequence homology have been indicated to bind to odorants with similar chemical structures [29,30]. Similarly, OR genes with high sequence homology across different species could also recognize similar odorant substances.

We observed that 21% of the OR clusters (n = 148) had genes that were common to all four species, and this type of cluster was the most common (Table 6). The second most common type of clusters contained genes common among mice, dogs, and pigs but not humans; this is consistent with the preferential loss of OR genes in the human genome. We found 171 of the 212 pig specific OR genes were functional genes, showing that the pig contains the largest number of unique OR genes among the species considered in this study. The number of clusters or subfamilies specific to pigs, humans, mice, and dogs was 61, 4, 39, and 19, respectively (Figure 3, Additional file 8). The presence of unique or common OR genes across different species reflects the maintenance and diversification of genes from common ancestors or the loss of genes within specific lineages during evolution, thus leading to OR subgenome diversity. Consistent with this, we found that the protein sequences of functional OR genes in pigs were highly similar (>70%) with those of OR pseudogenes of other species (Additional file 9).

**Table 6:** Number of common or unique olfactory receptor genes among pig, human, mouse, and dog olfactory receptor repertoires

**Species sharing the same OR gene clusters**	**Number of OR genes belonging to the species common clusters**			
	**Pig**	**Human**	**Mouse**	**Dog**
Pig, human, mouse, dog	341	179	255	228
Pig, mouse, dog	239	-	172	181
Pig, human, dog	78	61	-	62
Pig, human, mouse	82	37	55	-
Human, mouse, dog	-	38	46	45
Human, mouse	-	18	24	-
Human, dog	-	16	-	23
Pig, human	34	24	-	-
Mouse, dog	-	-	55	61
Pig, mouse	113	-	95	-
Pig, dog	138	-	-	96
Pig	212	-	-	-
Human	-	24	-	-
Mouse	-	-	131	-
Dog	-	-	-	45

Note: Sequences with more than 60% of amino acid sequence identity were clustered together. Outliers with significant sequence difference from the rest of ORs were excluded from the results.

A recent study in humans showed that a polymorphism in a region on chromosome 11 containing the OR genes OR51B5 and OR51B6 was associated with fetal hemoglobin concentration. This indicates that the elements within this OR gene cluster may play a regulatory role in gamma-globin gene expression [48]. The stereotypical mating posture of an estrus female pig when exposed to a compound in the saliva of boars is also mediated by the olfactory system [49]. Further studies on OR genes and their functional importance could elucidate phenotypes other than olfaction, such as reproductive or behavioral traits, that may be associated with OR gene clusters.

## Conclusions

We performed a genome level analysis of OR genes in the pig genome using conserved motif sequences specific to OR genes. Since the current pig genome assembly covers 99.9% of the pig genome, our result represents almost the entire OR gene repertoire of an individual pig genome. The pig OR gene family consists of 1,301 genes including pseudogenes, thus making it one of the largest known OR repertoires and suggesting an expansion of OR genes in the pig genome. The large number of OR subfamilies in pigs could contribute to the functional diversity of the olfactory system of pigs and allow pigs to recognize more diverse odorants than other animals.

## Competing interests

The authors declare that they have no competing interests.

## Authors’ contributions

Both DTN and KL carried out the bioinformatics analyses and classification of porcine OR genes, interpreted the data, and drafted the manuscript. HC, MC, MTL, and NS evaluated the results of the bioinformatics analyses. JHK, HGS, and JWO provided helpful ideas and discussion for the experiments. KL and THK were collaborators for the SGSC within Korea and played important roles in carrying out the project. CP was involved in project planning, discussion, and writing of the manuscript as a project principal investigator. This study was based on the swine genome sequencing results provided by the SGSC. All authors read and approved the final manuscript.

## Supplementary Material

Additional file 1 **Criteria for pattern recognition of pig OR genes using the PRATT program.** Table describing parameters and values for pattern recognition of pig OR genes. Click here for file

Additional file 2 **The pig OR gene coordinates in the pig genome assembly Sscrofa10.2.** Table listing positions of OR functional and pseudo genes in the pig genome. Click here for file

Additional file 3 **Comparison of the family and subfamily diversity of OR genes among pigs, humans, dogs, mice, and rats.** Table showing the results of comparative analysis of the number of classes, families, and subfamilies among five species including pigs, humans, dogs, mice, and rats. Click here for file

Additional file 4 **The number of OR gene members in OR subfamilies.** Table showing the number of OR gene members in each OR subfamily. Click here for file

Additional file 5 **Distribution of OR gene duplications in the pig genome.** Table showing the distribution of OR genes duplicated in the pig genome. Click here for file

Additional file 6 **The number of identical OR genes and their copy numbers in the pig genome.** Table listing the number of OR gene duplications and their copy numbers in the pig genome Sscrofa10.2. The maximum number of identical genes was three. Click here for file

Additional file 7 **Comparison of structural characteristics of OR gene clusters among five mammalian species.** Table listing number of clusters, number of genes per cluster, and number of clusters with only pseudogenes for pigs, humans, dogs, mice, and rats. Click here for file

Additional file 8 **Amino acid sequence similarity between the functional OR genes of pigs and OR pseudogenes of other species.** Table listing 19 pairs of pig OR functional genes and pseudo genes of other species with high protein sequence homology (>70%). Click here for file

Additional file 9 **The uncompressed image of Figure**3**.** The figure shows the uncompressed image of 706 clusters from 3,511 sequences of pig, human, mouse and dog ORs. Click here for file
